# Snoring: is a reliable assessment possible?

**DOI:** 10.1007/s00405-020-05813-2

**Published:** 2020-02-03

**Authors:** René Fischer, Thomas S. Kuehnel, Veronika Vielsmeier, Frank Haubner, Steffen Mueller, Christian Rohrmeier

**Affiliations:** 1 Department of Otorhinolaryngology, University of Regensburg, 93042 Regensburg, Germany; 2 Department of Otorhinolaryngology, University Medical Center Munich, Klinikum Großhadern, Marchioninistr. 15, 81377 Munich, Germany; 3 Department of Oral and Maxillofacial Surgery, University of Regensburg, 93042 Regensburg, Germany; 4 Faculty of Medicine, University of Regensburg, 93042 Regensburg, Germany; 5 ENT Medicinal Office, Bahnhofstr. 19, 94315 Straubing, Germany

**Keywords:** Snoring, Acoustic assessment, Bed partner, Annoyance, Snoring index

## Abstract

**Purpose:**

It is not easy to assess how severe and annoying a patient’s snoring is. Solid parameters are lacking; snorers cannot deliver a reliable self-assessment and it is uncertain whether bed partners’ statements can be relied upon. The purpose of the present study was therefore to investigate whether and how well snoring assessment based on acoustic parameters and bed partners’ reporting agree.

**Methods:**

In a double-blind, placebo-controlled study on snoring treatment, several acoustic parameters [snoring index (SI), percentage snoring time (ST), sound pressure level, sound energy, loudness, psychoacoustic annoyance and psychoacoustic snore score (PSS)] were measured in 18 subjects during 24 polysomnographies. Bed partners also assessed snoring annoyance and loudness as well as treatment outcome.

**Results:**

No correlation was found between the subjective annoyance caused by snoring and the acoustic parameters. Regarding perceived loudness, there was a moderate, significant correlation with loudness (*N*_5_) and PSS over the hour with the highest SI. SI, ST, LAeq and maximum sound pressure level dB(*A*)_max_ showed no significant correlation. After the intervention only mean sound energy LAeq over the entire night showed a significant correlation (*r*_s_ = 0.782; *p* = 0.022) with bed partners’ assessments. However, this result was not confirmed in the second control night.

**Conclusions:**

The non-existent or only weak correlation between bed partners’ ratings and objective parameters indicate that snoring severity should be evaluated with caution. Neither acoustic parameters, at least for one measurement over just one night, nor bed partners’ ratings should be used as the sole basis for snoring assessment.

## Introduction

Bed partners’ symptoms are the main reason why a lot of snoring patients seek treatment [1]. Partners of snorers experience problems initiating and maintaining sleep, have daytime tiredness and are not refreshed in the mornings roughly twice as frequently [2]. Snoring can cause arousals in the bed partner or will even wake up the partner who then has difficulty getting back to sleep [3, 4]. Troxel et al. demonstrated that fewer sleep disturbances are associated with greater marital happiness, hence a correlation exists between sleep and relationship quality [5]. 35% of couples have continual marital disputes because of snoring [6]. Therefore it is not surprising that over a quarter of bed partners of snorers sleep in separate bedrooms [2]. Treatment of the snorer can therefore help to improve the partner’s quality of life as well [7].

Assessing the severity of the snoring is problematic for physicians, especially since the snorer’s own judgment does not always agree with that of their bed partner [8, 9].

Snoring assessment usually involves questioning the partner. In studies, this is often done by means of visual analogue scales (VAS) [6, 9, 10] and after treatment it is not uncommon to simply ask whether the snoring has got better [11]. The most commonly used objective measure is the snoring index (SI), hence the number of snoring sounds per hour; less commonly other acoustic parameters are employed, such as percentage sleep time or total snoring time (ST) [11, 12]. Psychoacoustic parameters, such as annoyance, are also used [13]. The few studies available on this subject, however, report some variable results in terms of objective and subjective parameters [12, 14].

The purpose of the present study was therefore to investigate whether and how well the snoring assessment based on acoustic parameters correlates with reporting by bed partners. Data from a snoring treatment study were analysed for this purpose, with particular regard to perceived annoyance and loudness but also the perceived improvement as a result of treatment.

## Materials and methods

### Study procedure, study subjects

In a double-blind, placebo-controlled study to investigate the effect of Incobotulinumtoxin type A in habitual snoring (EudraCT no. 2011-001779-38) an assessment of the snoring was done by each subject’s bed partner (who had been a bed partner for at least 3 months). Shortly before the snorer underwent polysomnography (PSG), each partner had to answer the following snoring-related questions:

- *Question 1* “To what extent does your partner’s snoring disturb you?”
  The answer was to be rated on a scale from 0 (not at all) to 10 (extremely/unbearable).
- *Question 2* “How loud is the snoring?”
  Answer from 0 = no snoring, 1 = low, 2 = loud, 3 = audible in the next room to 4 = audible throughout the entire house.
- For the subjects who received a trial treatment, the following additional question was asked before the control nights:
  *Question 3* “Regarding your partner’s snoring, how would you judge the success of the treatment?” The answer had to be given on a scale from − 2 (snoring is much worse) to + 3 (snoring has disappeared).

Snoring was studied in 18 subjects (11 male) regularly snoring for at least 3 months with a mean age of 47.2 years (± 11.1; 24–69) and a mean BMI of 26.1 kg/m^2^ (± 3.5; 19.0–31.5). These subjects underwent a PSG (WatchPAT200, Itamar Medical Ltd., Caesarea/Israel) with digital recording of their snoring using an audio and acoustic analyser with class 1 microphone (XL2 with M2210, NTi Audio AG, Schaan/Liechtenstein). All the recordings were made in the university department under standardized acoustic conditions (structurally identical single room with identical furnishings, the same measuring set-up, microphone exactly 50 centimetres above the tip of the subject’s nose). Immediately before the measurements, a screening of sedatives and an alcohol test were performed which had to show no abnormalities (no sedatives, negative alcohol level).

A first PSG was performed on all 18 subjects. Of these, 10 subjects remained in the screening group. 8 subjects who fulfilled the inclusion criteria (including AHI under 10, BMI under 30) were included in the intervention group. These eight subjects underwent two further PSGs, where the mean interval to the second was 28 (± 3.7; 21–35) days and the mean interval between the second and third sleep studies was 62 (± 14; 49–91) days. The third PSG was made to see if a permanent change occurred. Of these eight subjects, two received a “treatment” (unilateral injection into the palate) with placebo and six received active treatment (Incobotulinumtoxin), both immediately after the first night.

### Acoustic analyses

Firstly the SI and the percentage ST were calculated from the acoustic parameters documented on a second-by-second basis. In light of a preliminary study (manual evaluation), sounds ≥ 38 dB LAeq (mean sound energy, *A*-weighted) were defined as snoring sounds. Secondly, the mean sound pressure level [(dB), *A*-weighted] and mean sound energy (LAeq) were calculated over the entire sleeping time.

For the hour with the highest SI, based on the audio files stored in WAV format (Windows PCM, 48,000 Hz, 32-Bit, mono) further calculations were done with dBSonic version 4.13 software (01 dB-Metravib, Limonest cedex/France). Mean and maximum sound pressure level [(dB), *A*-weighted] and the fifth percentile of psychoacoustic loudness (*N*_5_) were determined. In addition, psychoacoustic annoyance (PA) was calculated according to the formula of Zwicker and Fastl and the psychoacoustic snore score (PSS) according to Fischer et al., which indicate the annoyance of a sound and of a snoring sound, respectively [15, 16].

### Statistics

Microsoft Excel 2016 for Windows software (Microsoft Corporation) and SPSS Statistics 25.0 software (IBM Corporation, Armonk/USA) were used for the statistical analysis and creating the graph. Values below 0.05 were classed as significant.

The correlation between the answers to questions 1 (annoyance due to snoring) and 2 (loudness of snoring) and the objective acoustic parameters from the first PSGs on 18 subjects was investigated. Spearman’s rank correlation coefficient was applied because the answers to the questions were given on an ordinal scale and in order to avoid distortions caused by the fact that nights might differ widely in snoring severity (e.g. very loud).

Particularly in non-(severe) obese cohorts a dependence of the respiratory disorders on the supine position or REM sleep may exist. Such a dependence can be calculated with the Cartwright index modified by Steffen et al. [17]. This index was therefore calculated for each PSG. Using the Wilcoxon rank sum test, it was calculated whether there was a significant difference between the second and third PSG.

## Results

The results of the PSGs for sleep apnea parameters are listed in Table 1.

**Table 1:** Outcome data for sleep apnea parameters

	All PSGs	1. PSG	2. PSG	3. PSG
AHI	7.8 ± 7.6; 0–24	8.9 ± 7.7; 0–24	*7.2* ± *8.0; 0–20*	*5.9* ± *7.4; 0.6–22*
ODI	3.8 ± 4.4; 0.0–13	4.3 ± 4.3; 0.0–13	*3.7* ± *5.3; 0.0–13*	*2.6* ± *3.9; 0.0–11*
Supine-AHI	11 ± 11; 0.0–39	13 ± 11; 0.0–39	*10* ± *12; 0.0–32*	*7.4* ± *9.1; 0.3–26*
REM-AHI	11 ± 8.8; 0.0–30	10 ± 8.2; 0.0–28	*13* ± *11; 0.0–29*	*9.6* ± *9.2; 0.6–30*
Modified Cartwright index: supine	0.66 ± 0.31; 0.00–1.00	*0.74* ± *0.20; 0.30–0.98*	*0.65* ± *0.28; 0.00–0.90*	*0.75* ± *0.30; 0.08–1.00*
Modified Cartwright index: REM	0.41 ± 0.22; 0.00–0.80	*0.37* ± *0.19; 0.09–0.60*	*0.48* ± *0.25; 0.00–0.76*	*0.51* ± *0.19; 0.19–0.74*

The table shows the results of the polysomnographies (PSGs), each for all nights (for all 18 subjects) and separately for the individual nights (italic: data of the 8 subjects with several PSGs). In addition, the Cartwright index, modified by Steffen et al., is given, which shows whether supine or REM-associated sleep apnea is present [17]. Mean value ± standard deviation, minimum–maximum. *AHI* apnea–hypopnea index; events/h, *ODI* oxygen desaturation index; events/h, *PSG* polysomnography, *REM* rapid eye movement

For question 1 (annoyance due to snoring) all correlations showed no significant results (see Table 2). For question 2 (loudness of snoring) there was a moderate, significant correlation with the mean fifth percentile of loudness and with PSS, each in the hour with the highest SI. The acoustic values averaged over the entire night (SI, LAeq), the percentage ST and the maximum sound pressure level dB(*A*)_max_ showed no significant correlation.

**Table 2:** Correlation of primary nights with the answers to questions 1 and 2

	SI	ST	dB(*A*)_max_	LAeq	*N*_5_	PA	PSS
Question 1 (annoyance)							
*r*	0.098	0.397	0.077	0.223	0.112	0.020	0.047
*p*	0.71	0.12	0.77	0.39	0.67	0.94	0.86
Question 2 (loudness)							
*r*	0.241	0.088	0.399	0.195	**0.510**	0.464	**0.501**
*p*	0.34	0.73	0.10	0.44	**0.030**	0.053	**0.034**

Spearman’s correlation (= *r*) of the answers to question 1 (annoyance due to snoring) and question 2 (loudness of snoring) with the acoustic values was calculated in each case, *p* denotes the level of significance. Significant values are marked in bold

*SI* snoring index, *ST* percentage snoring time, *dB(A)*_*max*_ maximum sound pressure level, *LAeq* mean sound energy, *N*_*5*_ fifth percentile of loudness, *PA* psychoacoustic annoyance, *PSS* psychoacoustic snore score

**Fig. 1:**
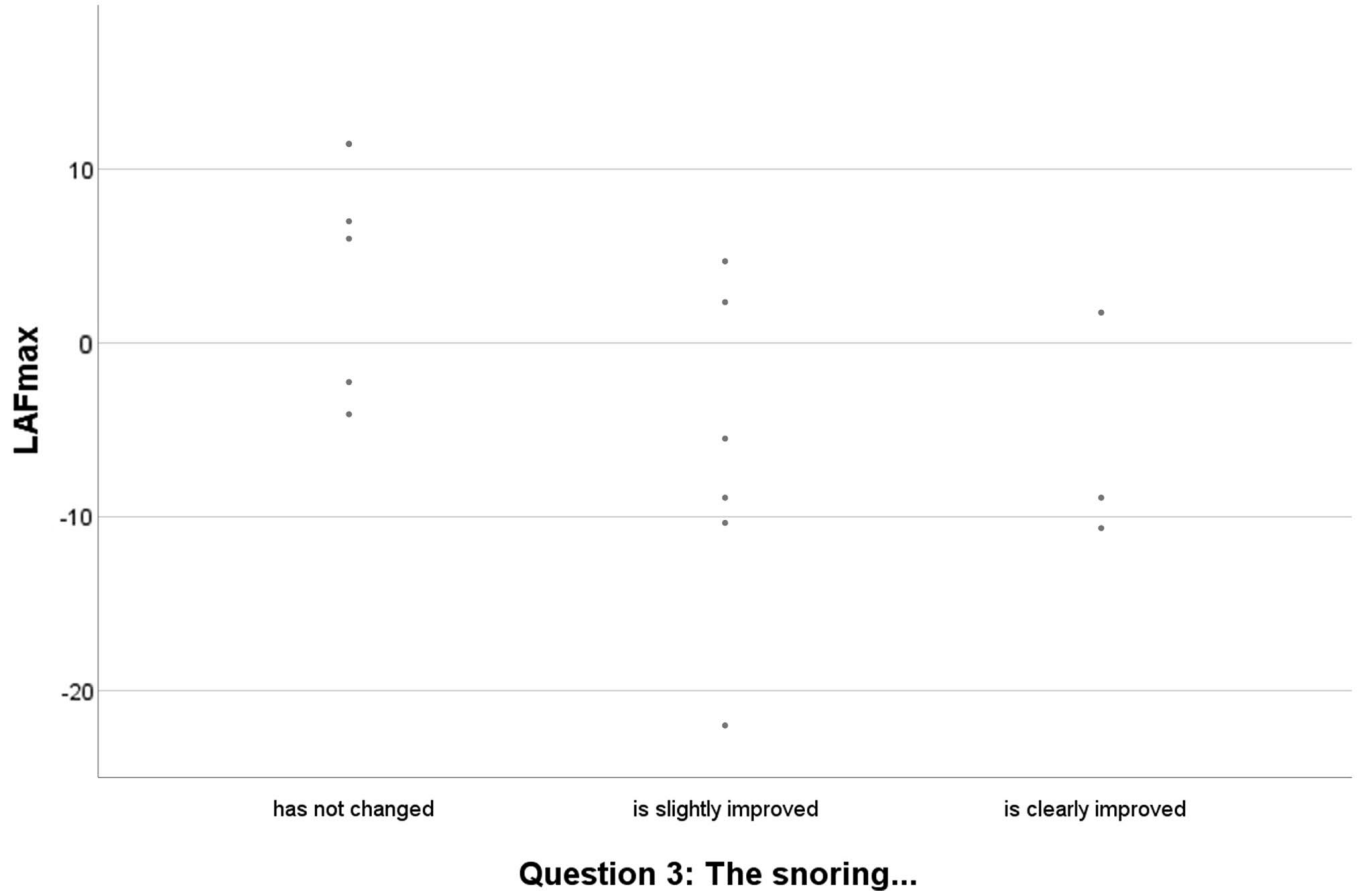
Assessment of treatment outcome. The graph shows the assessment of bed partners regarding treatment outcome before the second and third polysomnography in comparison with the mean sound energy level (LAF_max_) over the entire sleep time

After the treatment had been carried out, questioning of bed partners (question 3) and control PSGs (see Fig. 1) took place at two points in time. At the first time point 37.5% reported no change in snoring, 50% an improvement and 12.5% that the snoring had disappeared. In a correlation according to Spearman these results showed a significant correlation (*r*_s_ = 0.782; *p* = 0.022) only for mean sound energy LAeq over the entire night (see Table 3), but no significant correlation with the other parameters.

**Table 3:** Assessment of treatment outcome comparing the second and third polysomnography

	SI	ST	dB(*A*)_max_	LAeq	*N*_5_	PA	PSS
Second night							
*r*	0.235	0.394	0.196	**0.782**	0.574	0.391	0.665
*p*	0.58	0.34	0.64	**0.022**	0.14	0.34	0.072
Third night							
*r*	0.286	0.246	0.586	0.605	0.189	0.113	0.057
*p*	0.53	0.60	0.17	0.15	0.69	0.81	0.90

Spearman’s correlation (= *r*) of the answers to question 3 with the acoustic values was calculated, *p* denotes the level of significance. Significant values are marked in bold

*SI* snoring index, *ST* percentage snoring time, *dB(A)*_*max*_ maximum sound pressure level, *LAeq* mean sound energy, *N5* fifth percentile of loudness, *PA* psychoacoustic annoyance, *PSS* psychoacoustic snore score

At the time of the second control PSG 50% of bed partners reported no change in snoring, 25% an improvement and 25% an absence of snoring sounds compared with baseline before treatment. This time there was no significant correlation with any of the acoustic parameters (see Table 3).

There was no significant difference in the modified Cartwright index between the second and third PSG with respect to supine AHI (*p* = 0.40) and REM AHI (*p* = 0.67) in the Wilcoxon rank sum test.

## Discussion

It is a desirable goal to be able to assess snoring and the annoyance it causes to bed partners in a simple and reliable way, especially before making any treatment decisions. The results of the present study show, however, that currently common acoustic parameters and the reporting of bed partners do not deliver the same result. Even the loudness perceived by bed partners only agrees with a few acoustic parameters and only then shows a tendency to agree. Treatment-induced changes perceived by bed partners also do not correlate reliably enough with the objective results. This means that one cannot rely on reporting by the bed partner alone or solely the results of acoustic parameters for a satisfactory assessment of the risk–benefit relationship of potential therapeutic methods when treating the snorer.

Parameters used in this study were the percentage ST and the SI, which provides information about the frequency of snoring. There is no clear definition for SI measurement and calculation. Typically sounds above a certain threshold are recorded as snoring sounds and the number of such sounds per hour is calculated.

Caffier et al. showed that LAeq is the best parameter for distinguishing between snoring and non-snoring; in their study all snoring sounds were louder than 35 dB(*A*) [18]. We also found LAeq to be the best parameter in a preliminary study, but with a threshold of 38 dB(*A*).

Other values are partly used in other studies, which is because the measuring method employed, the distance to the microphone or the room where measurements are taken, for example, will all influence the result [19, 20]. The patient’s position also plays a role: turning over in bed can cause a difference of up to 3 dB [21].

At the same time, parameters were analysed in the present study which provide information about the loudness of snoring. These were the *A*-weighted mean sound energy LAeq over the entire sleeping time, the maximum sound pressure level dB(*A*)_max_ and for the hour with the highest SI the fifth percentile of loudness (loudest 5% of sounds). It was shown in studies that the fifth percentile of loudness reproduces the perceived loudness of sounds very well [15].

To ascertain the annoyance of snoring, PA and PSS were each calculated for the hour with the highest SI. Studies have shown that the annoyance of sounds correlates well with the level of these parameters generally and specifically for snoring sounds in the case of PSS [13, 15, 16].

It is interesting, however, that the acoustic parameters did not show the assumed correlation with the subjective reports of snorers’ partners. It is worth noting that PSS was established specifically for snoring sounds and thus probably performs rather better than PA. However, PSS was developed in the acoustic laboratory based on assessments by “unaffected” people [16]. According to the present results, it seems inappropriate to translate such acoustic parameters to a couple relationship for the purpose of assessing snoring.

In one of the few studies available on this subject Hoffstein et al. showed that, even between the evaluation by sleep laboratory staff, who directly observe snoring in their assessment, and objective parameters, only a partly weak correlation exists. In their study, for instance, the correlation with SI at *r* = 0.68 (*p* < 0.01) was in the middle range and that with dB_mean_ at *r* = 0.34 (*p* < 0.01) in the lower range [12].

Treatment outcome as perceived by bed partners also did not correlate with the objectively measured parameters in the present study. Miljeteig et al. came to a similar result. They studied patients by PSG before and after uvulopalatopharyngoplasty (UPPP) [14]. The objective measurements showed no significant differences with regard to AHI, SI and mean and maximum sound pressure level. In the questionnaires completed by the patients, however, 78% stated that their snoring was reduced and 79% reported an improvement in their sleep. Interestingly, 68% of their bed partners stated that their sleep was disturbed by snoring before the operation, but only 20% postoperatively.

Hence there must be other factors that influence the subjective assessment of snoring. It is known from noise research that non-acoustic influences are also relevant to an evaluation of noise [22, 23]. In the annoyance assessment, only 10–20% of the perceived annoyance could be explained by the sound pressure level [22].

Relevant factors come from various areas: the personal factors include individual noise sensitivity. Ellermeier et al. showed that the latter plays a significant role in the assessment of sounds [24]. Social status or, for example, working conditions (night shift) similarly play a role [25]. Furthermore situation-dependent factors have an influence as well as the relationship to the “noise source”, personal perception and possible expectations [25]. For example, people who benefit from a new rail link perceive the noise it causes as far less annoying [22]. By extension, that means the relationship between snorer and bed partner seems to have a significant part to play.

It is fascinating in this respect that the bed partner and the snorer do not always agree in their snoring assessment, even though snorers actually get their main information from their bed partner. Virkulla et al. showed that a correlation of only *r* = 0.51 existed for snoring intensity and only *r* = 0.35 for snoring duration (both significant) [6]. Hoffstein et al. also found only a very moderate agreement of 0.22 (Cohen’s *κ*) for these two parameters [12].

Night-to-night effects might be another significant reason for the lack of correlation in the present study. Carthcart et al. studied how much snoring of 20 snorers varied in four different nights [26]. They found distinct night-to-night differences. For instance, a standard deviation was 104 snoring sounds per hour for SI and 4.1 dB for loudness L1. According to the authors, the variance attributable to a normal night-to-night variation is 22% for L1 and even 26% for SI. These are relatively high values.

The data recorded in the present study are of high quality. The subjects were carefully selected and each subject underwent a drug and alcohol test before each night, blood levels were measured and the BMI was checked in order to minimise other factors that might influence snoring. Weaknesses of the study include that only one night per subject was measured before and after the treatment in the study period and that the overall number of subjects was low.

However, the non-existent or only weak correlation between the ratings of bed partners and the objective parameters indicates that the severity of snoring should be evaluated with caution. Neither the acoustic parameters, at least for one measurement over just one night, nor the reporting of bed partners should be used as the sole basis for assessing snoring. Instead, one should build up a complete picture which includes a personal impression of the snorer, of the snorer’s bed partner and the perceived quality of their relationship.
